# Optimizing the technological and informational relationship of the health care process and of the communication between physician and patient– Factors that have an impact on the process of diagnosis from the physician's and the patient's perspectives

**Published:** 2011-05-25

**Authors:** VL Purcarea, DG Petrescu, IR Gheorghe, CM Petrescu

**Affiliations:** * ‘Carol Davila’ University of Medicine and Pharmacy, Faculty of Dentistry, BucharestRomania; ** Academy of Economic Studies, BucharestRomania

**Keywords:** diagnosis process, information technology, electronic medical records, telemedicine

## Abstract

**Objective**: the optimization of a diagnosis process and fluency in the Health Care sector in Romania. A key to discover this complex process was to determine a correlation between the physicians and the use of information technology, on one side and the patients' perspective on the other.

**Hypothesis**: Integrating information technology in a physician's activity will lead to lower costs and less time spent while diagnosing patients. Using the electronic medical records and introducing a unified database with the patients' medical histories will make the process of diagnosis easier.

**Methods**: We studied the diagnosis from the point of view of 304 patients in a public hospital and 320 physicians working there.

**Results**: We believed that time and accessibility to different physicians makes the diagnosis process a burden for a patient and implicitly lead to dissatisfaction with health care services. We supposed that the burden of diagnosis for physicians comes from the lack of Internet connection and computer usage knowledge. We have found out that most physicians know how to use the computer at an intermediate level and have access to Internet, online journals and databases and do not use emails to a higher extent to communicate to other specialists, but do not rely entirely on the electronic medical records. Most physicians think that it is not technology, which stands in the way of proper and fast diagnosis but the financing and the paper work from the Romanian health system. Solutions that might be taken into account to entirely motivate physicians to use electronic medical records are:

1. Adjustments can be made to the computer software interface in order to make the design more consistent (to eliminate the paper forms) and user friendly.
2. Physicians can be provided with more training and knowledge.

## Introduction

This paper is the second part of a research that studied the application of Preventive Medicine in Romania and the communication relationship between patients and doctors. Its factors make the Health Care diagnosis heavier and more important.

The first part of this study, called the preliminary study, described the problem of preventive medicine and was presented in Journal of Medicine and Life, vol. 4, no. 1 (January–March 2011). [1] The conclusion of the study was that the youngsters who made up the sample group were not influenced in any way by the concept of preventive medicine; although they had a healthy lifestyle. The whole study was also presented in HICT 2011–International Forum on Health Care and Information Communication Technology, in Barcelona, Spain, on 8^th^–10^th^ of March 2011. [2] 

The second part of this study explains and analyses the process of diagnosis both from the patients' perspective and from the point of view of the physicians who are involved in the doctor–patient process of care and communication. 

It is important to mention that the doctor-patient relationship represents the center of the medical practice, and, in the same time, it is essential in the process of diagnosis and in the provision of the quality medical services. When talking about the tracking and optimizing of the technological and informational relationship of the health care process, the main factors that influence the process of diagnosis should be taken into consideration. The three most important goals in disease diagnosis are:

- speed;
- cost;
- accuracy;[3]

The diagnosis represents the process of identifying a disease or disorder in a person by examining the person and studying the results of the tests. It is based on information that the physicians obtain from the patient's perceptions of his symptoms, medical history, family history, the environment in which the patient lives in and other relevant facts. Afterwards, the physician narrows down the information obtained from the examination of the patient and does some medical tests. Then he concludes by giving a diagnostic. All the processes, in different stages, are influenced by several factors.

So, the diagnostic decision–making is based on the experience and hypothetic–deductive reasoning of the physician. [4] When dealing with a diagnostic uncertainty, a doctor can either gather more evidence or treat the patient. [5]

There are three different cases of medical diagnosis errors due to the lack of several materials or some factors happening at the time the health care service is being delivered.[6]

1. The false–negative case in which a patient, who in reality has a disease, is diagnosed as disease free.
2. The false–positive case in which a patient, who in reality does not have the disease, is diagnosed as being ill.
3. The unclassifiable case in which the prediction system cannot diagnose a given case due to insufficient information or knowledge.

The conclusion that can be drawn is that the process of diagnosis can be influenced anytime, by several factors.

**Figure 1:**
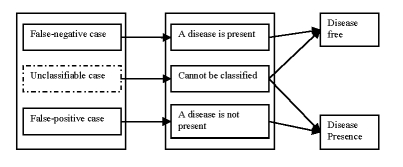
Medical diagnosis errors

One of the factors, which the study was based upon, was the lack of technology in Romania and the lack of an organism (institution, device, etc.) able to measure and detect the weak spots of the healthcare system. 

## Background

In 2010, the European Commission has analyzed and evaluated the degree of computerization of the healthcare services in all the states that are members of the European Union. The project included the analysis of every country according to some criteria :

- the applicability level of the strategies and politics used in the present computer system;
- the existence of a documentation regarding these strategies and politics from a legislative point of view; which are the proposed measurements and which are the stages necessary to be completed while making the analysis;
- the involving level of the Ministry of Health;
- the degree of using the electronic charts and telemedicine;
- the interoperability degree between the actors in the health system

According to these criteria, there is no official institution in Romania willing to evaluate the aspects requested by the European Commission. Starting with 1992, an elaboration of a documentation regarding an e–health strategy, has been developed, but, until present, it has stopped in the project state, although, from a legislative point of view, in the 95/2006 law regarding the reform in health, it was mentioned that the Ministry of Health will implement an integrated computer system, comprising information regarding all the communicable diseases, emergency care, information about the hospitals, physicians, etc. What was also mentioned was the inclusion of the health cards, which have finally started to be included in the health system at the beginning of 2011.  

Therefore, at the beginning of 2011, the project ‘Unique Integrated Computer System of the National Health Insurance House’ has started developing. In the first phase, the health card will be made, then the electronic health charts of the patients, the e–prescriptions, etc. A relevant documentation regarding this project does not exist because the data are still collected and filtered, in order to guarantee an accurate and precise service. 

## Aim

The optimizing of the process of diagnosis and the insurance of the health services accurate providing in Romania, are made by evaluating some factors that influence those process. One of the factors is represented by the computer systems, or more precisely by the lack of computer systems. 

The diagnose processes can take for months, according to the reaction of some patients to certain stimuli as well as to their medical evolution. The determination of the complex relationship that exists between the information technology and the physician is important, because it envisages the way the working flux can be optimized, so that it improves the productivity of the work done by the physician. This way, the integration of the information technology in the medical activity will lead to lower costs and the reduction of the time spent to diagnose the patients. 

## Meth

The study was mad on a sample group of 304 patients and 320 physicians in a public hospital, in Romania. The diagnosis process was analyzed while taking into account the opinions of the two sample groups. 

Accordingly, the patients have decided that the factors, which make the diagnosis process mach harder to be accomplished, are the following:

- the time they have to wait in order to be consulted by a specialist, as well as the time necessary for an analysis.
- the accessibility to a family physician, specialists, home medical care, emergency medical care, etc.

The hypotheses are the following:

- The patient considers time an impediment in the process of diagnosis.
- The patient considers the accessibility to health services an impediment in the process of diagnosis.
- The physicians consider the absence of technology and electronic resources an impediment in the process of diagnosis.

**Figure 2:**
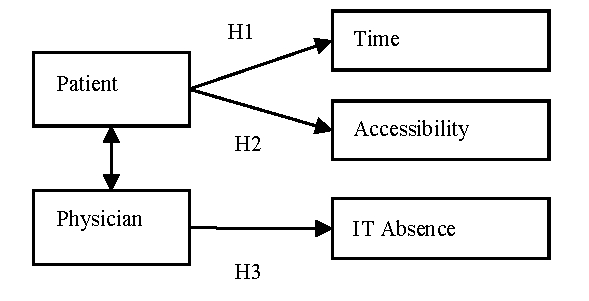
The hypotheses of the model

We consider the time and accessibility to the main health services essential from the point of view of the patient. The accessibility of the patients to the health services, which comprise the above–mentioned indicators, was measured with the help of Likert scale. 

The physicians included in this study are primary physicians and residents (last year) with different specializations, most of them having 15–20 years of medical practice or even more. 

In the physicians' case, the determinant factors which can make the process of diagnosis harder to accomplish are many, but we consider that the main factor is the lack of the Internet connection or the lack of a database with information regarding the patients and last, but not least, the impossibility of stocking the data about the patients. 

The results were obtained by using Microsoft Excel 2007 and 13.0 version of the SPSS software.

The correlations between the variables have been made by using the Pearson test of the SPSS software. The Pearson coefficient can take any values, which show the intensity and the direction of the connection. Therefore, if the value of the connection between two variables ranges or is close to +/– 0.6 si +/– 0.8, then there is a powerful positive or negative correlation. If the value of the connection is situated between +/– 0.1 si +/– 0.4, then, this is a weak correlation.

## Results

A panel of 304 patients and 320 physicians has been surveyed. What should be taken into consideration is the fact that the panel of the patients was made up of adolescents with the average age of 19 years old. 

Out of 304 patients, 293 had a valid answer. Out of 293 patients, only 37.5% of them spent time before seeing a specialist less than an hour and the vast majority (41.8%) spent between one and three hours until seeing a specialist.

**Figure 3:**
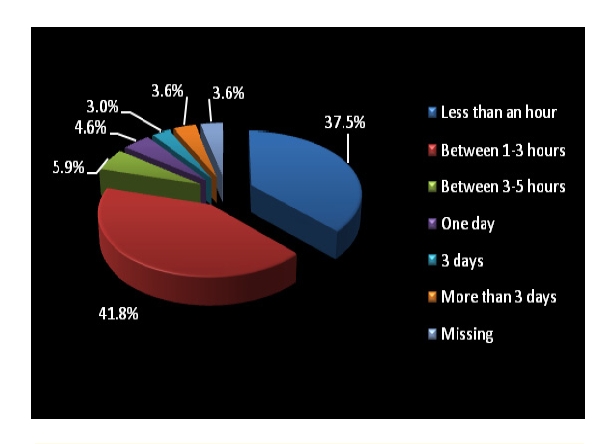
Time spent before seeing a physician

Out of 304 patients, 288 answered. For routine consultations, the diagnosis was made on the same day, in less than an hour, in 44.4% of the cases. 

**Figure 4:**
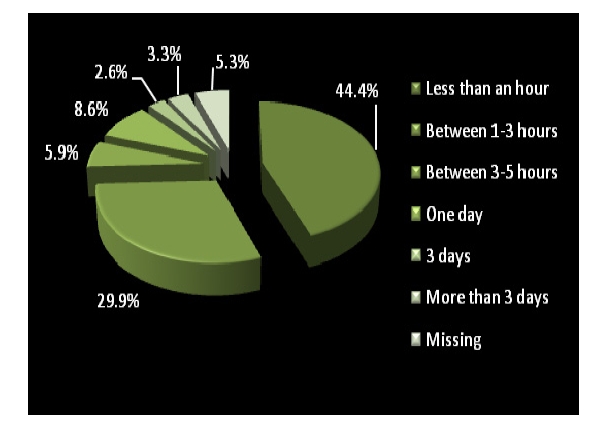
Time spent waiting for an easy diagnosis in a routine consultation

Out of 304 respondents, 291 answered. The diagnosis that required an analysis and other interventions was given in 3 days or more. Only 8.9% of the physicians made the diagnosis in less than an hour.

**Figure 5:**
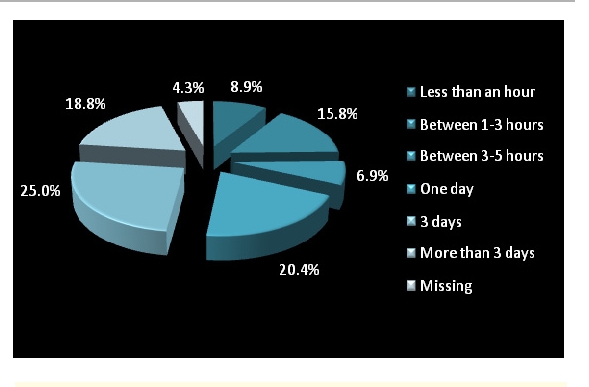
Time spent waiting for a diagnosis that required interventions and analysis

Out of 304 patients, 300 answered. 51% of the patients did not have a medical history and 38.8% did not know of an existing one.

**Figure 6:**
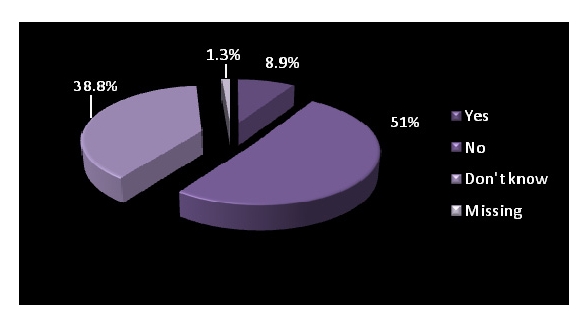
Electronic medical history

Out of 304 respondents, 295 answered. Most of patients agreed and strongly agreed to have access to family physicians. 

**Figure 7:**
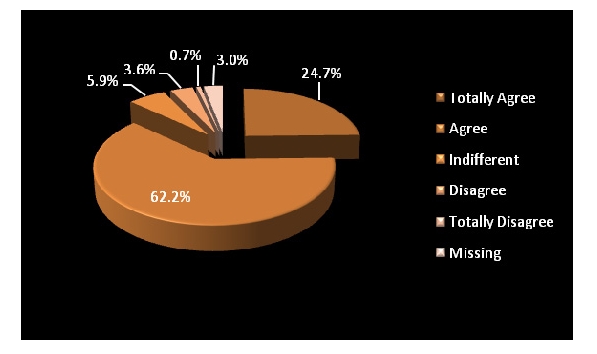
Accessibility to a family physician

Out of 304 patients, 292 answered. 58.2% of patients have access to professional care.

**Figure 8:**
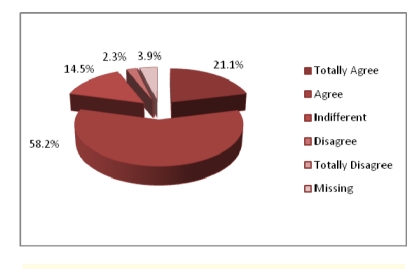
Accessibility to a primary physician

Out of 304 patients, 287 answered. Most of them have access to home health care services.

**Figure 9:**
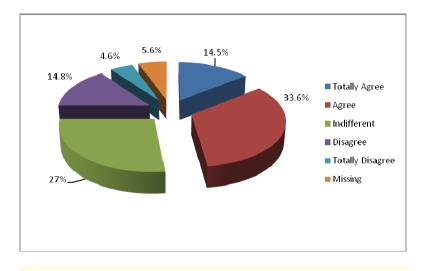
Accessibility to home health care services

Out of 304 patients, 5.3% of them did not answer. 42.8% agreed they have access to health care services in case of emergency and only 3.0% totally disagreed to not having access to these services.

**Figure 10:**
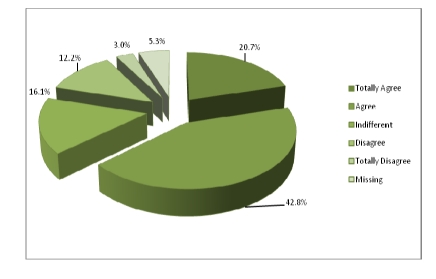
Accessibility to health care services in case of emergency

Out of 350 questionnaires sent to physicians, 320 returned. Most of the physicians from the panel have Internal Medicine as a specialty– 26.6%, followed by the ones with Surgery as a specialty.

**Figure 11:**
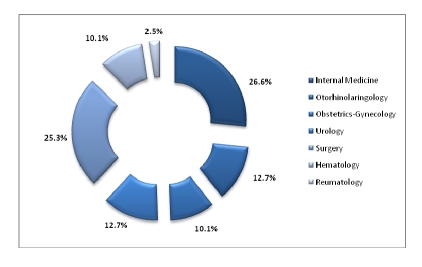
The specialties of the physicians

Being primary physicians, most of the respondents have practiced medicine for 15–20 years or more.

**Figure 12:**
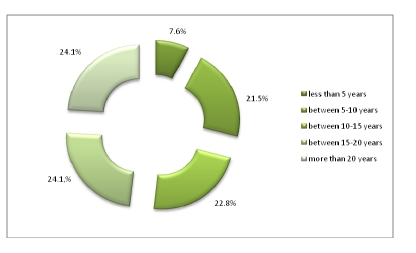
The practice of medicine (university years not included)

Out of 320 physicians, 74.7% answered they have access to technology and Internet in the health care institution they work in.

**Figure 13:**
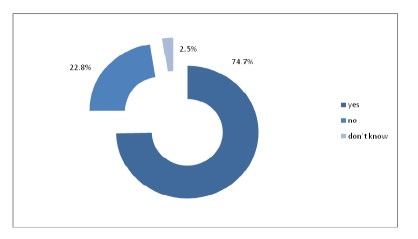
The health care organizations have access to technology and internet

Even if most of physicians have access to electronic medical records and they use the computer to write medical forms, all of them answered that the recipe given to the patients is manually written.

**Figure 14:**
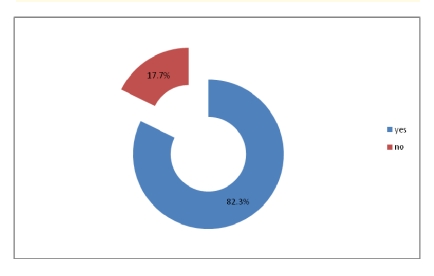
Physicians have access to electronic medical records

**Figure 15:**
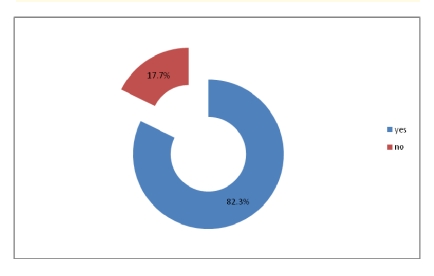
The physicians' computer usage to write medical forms

Most physicians do not use electronic means such as the e–mail or other platforms to exchange information, neither with other physicians nor with patients, but they use the telephone to talk with to the other specialists.

**Figure 16:**
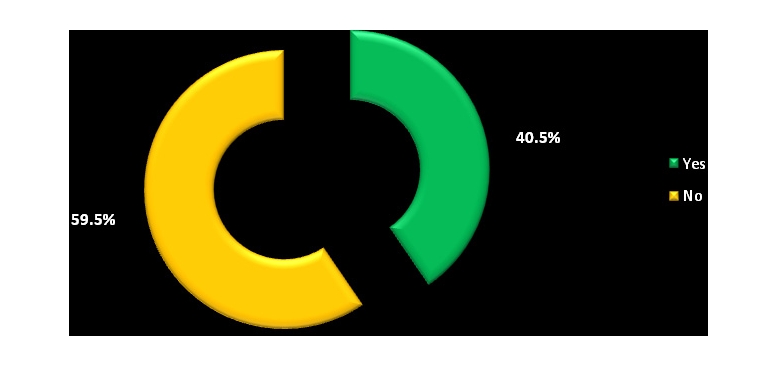
The use of computers in exchanging information with the other health care specialists

**Figure 17:**
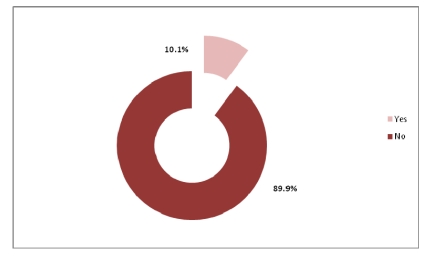
The use of e–mail in exchanging information with patients

**Figure 18:**
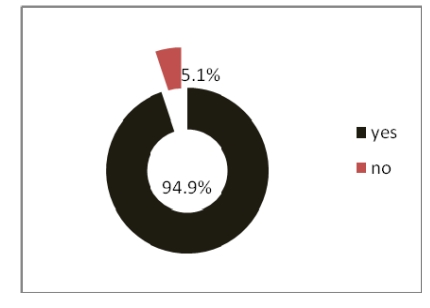
Telephone use in communication with the other specialists

Regarding the level of computer use, out of 320 physicians, 51.9% are situated at an intermediate level.

**Figure 19:**
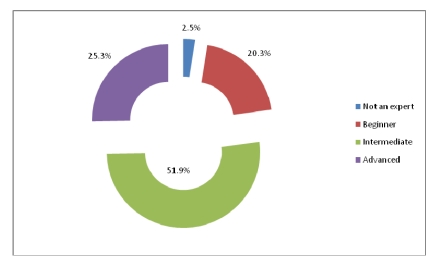
The level of PC knowledge

Out of 320 physicians, 70.9% chose a combination between paper and electronic tables to write the patient forms and used electronic archives for patient information.

**Figure 20:**
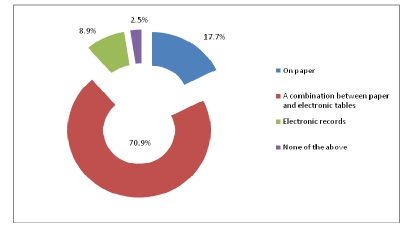
The way the patient forms are written

**Figure 21:**
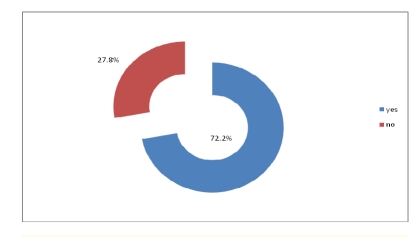
Using electronic archive for patient information, medical history

Most physicians do not get electronic help from the other physicians and do not use telemedicine either, but a percentage of 46.8% have access to online journals and medical databases and 39.2% have either a website or a blog where to post their research.

**Figure 22:**
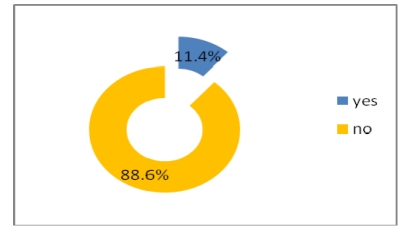
Electronic help in health decision–making

**Figure 23:**
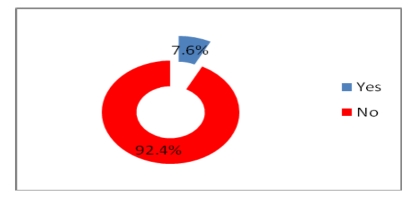
Telemedicine

**Figure 24:**
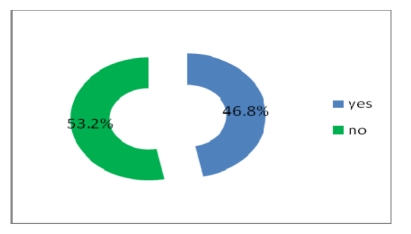
Online access to journals and medical databases

**Figure 25:**
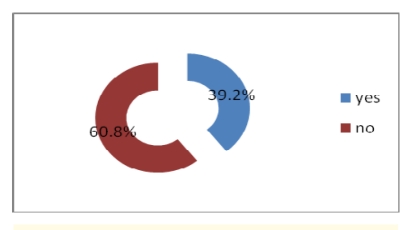
Having a web site or blog where physicians post research results, health care events

From the physicians' point of view, the process of diagnosis becomes heavier because of the financing of the health care system (20.5%), the bureaucracy (16.6%) and missing the proper facilities, in which patients to be treated properly (12.2%).

**Figure 26:**
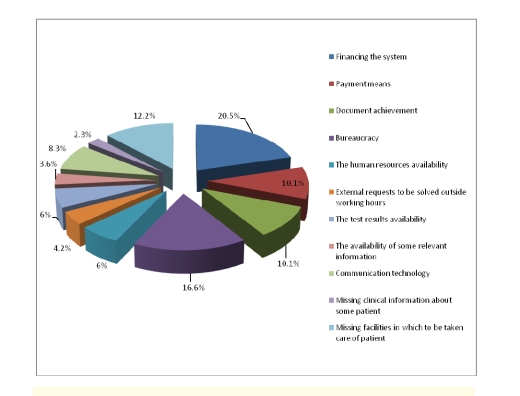
Factors that lead to a heavy diagnosis from the physicians' perspective

## Discussions

The second part of the study included elements of measuring the use of IT in the doctor–patient relationship, together with its factors that contribute to making the process of diagnosis harder to accomplish from both the physicians' and the patients' perspectives. 

The panel of patients was made up of 304 respondents, with the average age of 19, from Romania. Accessible routine consultations are not a burden for most of the patients; they do not spend a lot of time before being consulted by a physician. Most of the patients also agreed and strongly agreed to have access to family and specialized physicians, as well as to home and emergency healthcare.

The panel of physicians was made up of 320 respondents, the vast majority having practiced medicine for 15–20 years or more, and had Internal Medicine as their specialty.

Most of the physicians had access to technology, Internet and electronic medical records even if the recipe was still manually written.

Most physicians use electronic forms to enter patient information and use computers to exchange information with the other health care specialists but do not use the e–mail and the Internet in exchanging information with patients, or any other electronic means. They also know how to use the computer at an intermediate level.

While consulting patients, physicians use a combination between paper and electronic tables and, they also use electronic archives for patient information or medical history.

Most physicians do not have electronic help in health decision making, and do not use telemedicine, but they have access to journals and medical databases.

From a physician's perspective, the main causes that stand in the way of the process of diagnosis are the financing of the health care system, the bureaucracy and the improper facilities to take care of the patients.

Several correlations were made by using the Pearson coefficient, but none was found relevant for our study. No correlation was found between the specialty of the physicians and the PC knowledge (r=0.06, p<0.01). Likewise, between the fact that physicians use electronic forms and have access to several online information such as specialized dictionaries and journals (r=–0.03, p<0.01).

In conclusion, patients consider time to be a factor that makes the diagnosis heavier from their perspective, but they easily have access to healthcare services when in need.

Physicians do not consider the absence of technology a factor that makes the diagnosis a long–term process and they do not communicate with other patients or physicians through electronic means.
